# Successful implantation of S‐ICD using the intermuscular two‐incision technique in a patient with severe pectus excavatum

**DOI:** 10.1002/ccr3.5143

**Published:** 2021-11-25

**Authors:** Fabrizio Guarracini, Massimiliano Marini, Mattia Strazzanti, Roberto Bonmassari, Stefano Guarracini, Michele Di Mauro, Andrea Droghetti

**Affiliations:** ^1^ Department of Cardiology S. Chiara Hospital Trento Italy; ^2^ Department of Cardiology "Pierangeli" Hospital Pescara Italy; ^3^ Cardio‐Thoracic Surgery Unit, Heart and Vascular Centre Maastricht University Medical Centre (MUMC) Maastricht The Netherlands; ^4^ Thoracic Surgery Division ASST Mantova‐Cremona Mantua Italy

**Keywords:** pectus excavatum, serratus anterior plane block, subcutaneous implantable cardioverter defibrillator, two‐incision technique

## Abstract

A patient with severe pectus excavatum, dilated ischemic heart disease, and indication for defibrillator implantation for primary prevention of sudden death underwent successful ultrasound‐guided serratus anterior plane block and parasternal block with intermuscular two‐incision technique implantation with no complications. At follow‐up, all the parameters resulted stable with excellent signal sensing.

## Graphical abstract

In case of severe pectus excavatum, an ultrasound‐guided serratus anterior plane block and parasternal block with intermuscular two‐incision technique implantation can be useful.

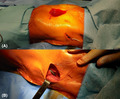

## 1 INTRODUCTION

Subcutaneous implantable cardioverter defibrillator (S‐ICD) is an alternative to transvenous ICD. It does not require implantation of transvenous or epicardial leads, preventing all related complications. It proved to be a safe and effective system for both primary and secondary prevention of sudden cardiac death.^1^, ^2^ Main limitations of S‐ICD are the inability to provide anti‐tachycardia pacing (ATP) for ventricular tachycardia and limited bradycardia pacing support (only 30 seconds post‐shock pacing). The implantation procedure has evolved over the years, and recently, a standardized approach with ultrasound‐guided serratus anterior plane block and parasternal block combined with the intermuscular two‐incision technique was introduced with the purpose of reducing local anesthesia, general sedation, and pain levels.^3^, ^4^, ^5^ Implantation of S‐ICD in patients with pectus excavutum is a challenging procedure due to the anatomical alterations of the structures that require adequate positioning of the defibrillation catheter to obtain satisfactory signal detection and defibrillation margin. In literature, there are isolated experiences describing the feasibility of this challenging procedure.^6^, ^7^, ^8^ In this report, we describe the case of a patient with severe pectus excavatum, in which the S‐ICD implantation procedure was accomplished by means of the intermuscular two‐incision technique with ultrasound‐guided nerve block to provide anesthesia/analgesia.

## 2 CASE REPORT

**FIGURE 1:**
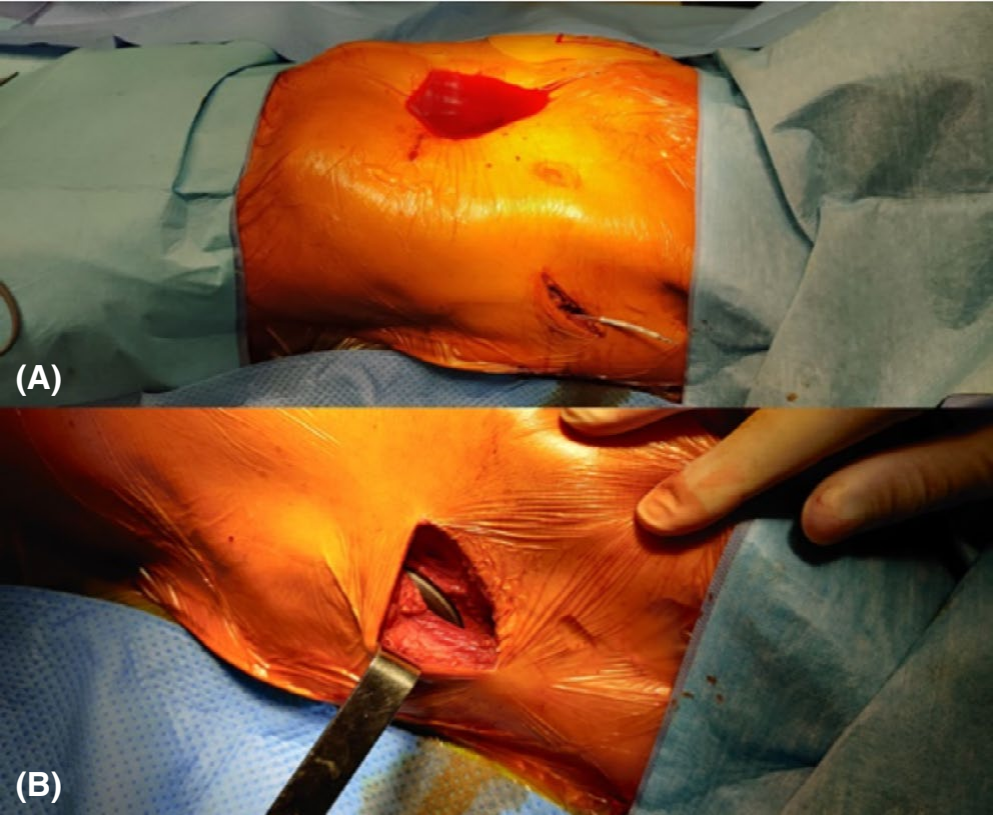
Two‐incision technique. The chest deformity is highlighted by the fluid (A). Intermuscular device placement (B)

**FIGURE 2:**
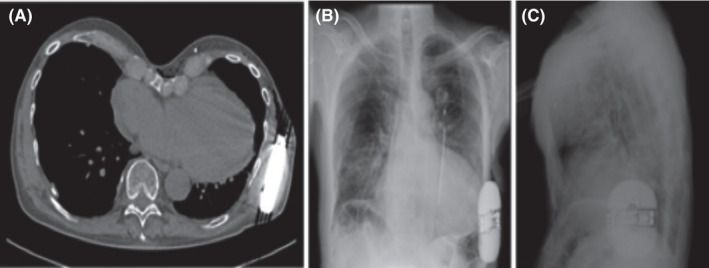
CT image (A), postero‐anterior (B) and lateral (C) chest radiography after S‐ICD implantation. Note how the severity of pectus excavatum changed the position of “cardiac area” inside the mediastinum

**FIGURE 3:**
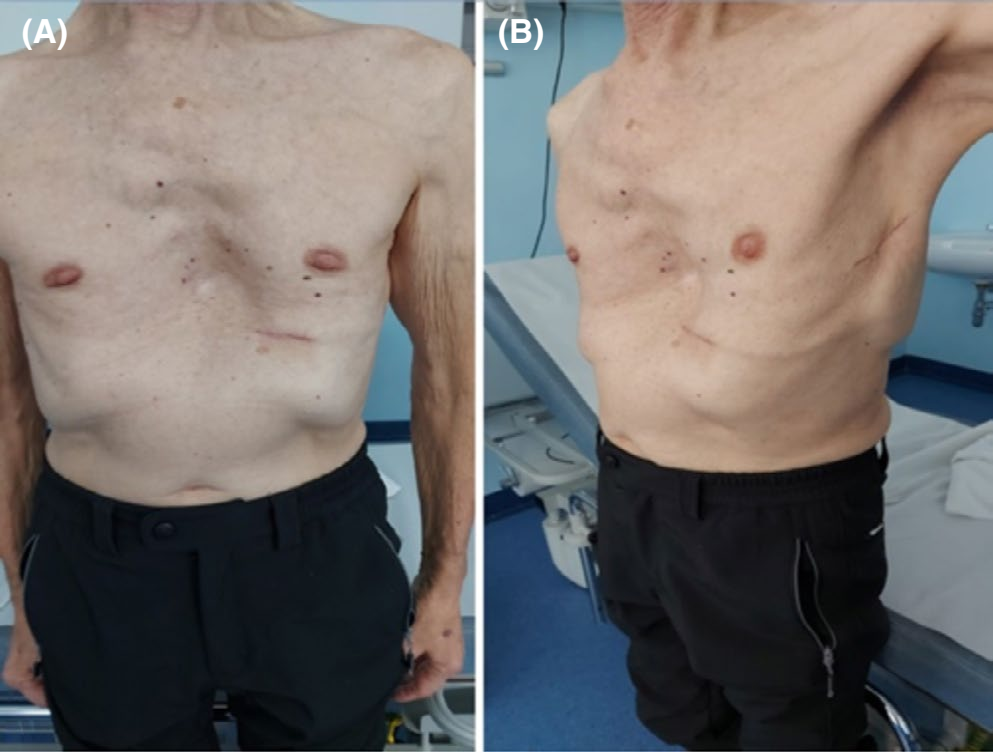
Evolution of surgical wounds at one month of follow‐up (A, B)

A 66‐year‐old male patient with ischemic heart disease and severely reduced left ventricle ejection fraction reduction (25%), non‐sustained ventricular arrhythmias, and severe pectus excavatum (Haller Index 5.2) was evaluated for S‐ICD implantation (Emblem MRI S‐ICD A219, Boston Scientific) for primary prevention of sudden cardiac death according to current guidelines.^9^ The absence of any anti‐bradycardia pacing or cardiac resynchronization therapy indication was verified. The impact of the chest deformity was carefully evaluated. The pre‐procedural electrocardiographic screening was performed, as suggested by the manufacturer, and it confirmed the adequacy of the primary and secondary vector signal, whereas it showed very low signal with the alternative vector in the left parasternal position. Before the implantation, serratus anterior plane block and parasternal block under echo guidance were performed according to the method previously described in literature.^3^, ^4^, ^5^ In particular, the plane between intercostal muscles and serratus anterior muscle was reached and bupivacaine was injected, blocking the signal of the lateral cutaneous branches. During the implantation, conscious sedation was provided by continuous infusion of remifentanil administered by a calibrated infusion device into a dedicated intravenous line. A two‐incision intermuscular technique with no cranial incision was completed (Figure 1), and defibrillation lead was successfully placed parallel 1 cm to the left of the sternal midline with the distal sensing electrode localized adjacent to the manubriosternal junction. The proximal sensing electrode was positioned 2 cm above the xiphoid process, across the chest deformity. The generator was positioned on the lateral chest wall, creating a pocket between the serratus anterior and the latissimus dorsi muscles. At the end of the procedure, defibrillation testing induced ventricular fibrillation that was successfully terminated by a 65 J shock. The shock impedance was 68 Ohms. Time from initial detection to the shock delivery was about 11.5 seconds. At the end of procedure, a conditional zone at 200 beats per minute (bpm) and a shock zone above 250 bpm was programmed. The procedural time was 54 minutes, and there were no complications. Good lead position was confirmed the day after the procedure by chest X‐ray with radiographs in two projections and by CT scan, performed for a suspicious pulmonary nodule without consequences (Figure 2). The day post‐implantation the patient underwent S‐ICD programming optimization and the primary vector was selected. Thus, the patient was discharged. At one‐month follow‐up, patient was asymptomatic, wound healing was normal (Figure 3) and no arrhythmic events were revealed at the S‐ICD interrogation.

## 3 DISCUSSION

To our knowledge, this is the first experience of successful S‐ICD implantation in a patient with severe pectus excavatum, performed by combining the intermuscular two‐incision technique with an ultrasound‐guided nerve block strategy for anesthesia/analgesia.

The intermuscular 2‐incision approach for S‐ICD implantation was shown to be safe and to result in better cosmetic outcomes and shorter procedural times,^3^, ^4^, ^5^ as well as in a very low risk of conversion failure according to the PRATORIAN score.^10^ Recently, the feasibility and efficacy of ultrasound‐guided serratus anterior plane block to provide anesthesia/analgesia during S‐ICD implantation was demonstrated.^5^ In comparison with a typical approach for local anesthesia and sedation, it was shown to be associated with lower pain levels, reduced need for sedation, and shorter procedure durations. Moreover, the addition of ultrasound‐guided parasternal block was shown to provide a reliable block of both the anterior and the lateral rami of the intercostal nerves, to ensure complete anesthesia of the anterolateral chest wall, providing a reliable anesthesia and analgesia for S‐ICD implant procedure.^4^

In the present case, despite the severe anatomical deformity of the sternum, we succeed in tunnelling the lead using the 2‐incision technique, thus avoiding the superior parasternal incision. Nonetheless, the patient anatomy conditioned the parasternal tunnelling of the lead which was more cranial and lateral than normal. To compensate for the forced vector correction, we decided to position the S‐ICD generator more caudal and posterior than usual (see Figure 3B). Despite the non‐standard positioning of the electrode and the generator, the system was found to be effective at the conversion test and the recommended safety margin of 15J was verified.

In conclusion, paying attention to the positioning the system, the intermuscular two‐incision technique with ultrasound‐guided nerve block seems feasible for S‐ICD implantation even in cases of patients with severe chest deformities.

## 4 LEARNING OBJECTIVES

- To confirm the feasibility of S‐ICD implantation in patients with severe chest deformities.
- To describe the implantation technique and the anesthesia/analgesia strategy that allowed successful implantation.

## CONFLICTS OF INTEREST

Dr. Andrea Droghetti is a consultant for Boston Scientific. The other authors declare that they have no conflicts of interest.

## AUTHOR CONTRIBUTIONS

Fabrizio Guarracini, Massimiliano Marini, Mattia Strazzanti, and Roberto Bonmassari involved in acquisition of data and drafting of manuscript. Stefano Guarracini, Michele di Mauro, and Andrea Droghetti involved in drafting of the work. Roberto Bonmassari, Andrea Droghetti, and Michele Di Mauro involved in substantively revising the final version.

## ETHICAL APPROVAL

The study was realized according to Helsinki Declaration, and all the ethical committees were respected.

## CONSENT

The authors have confirmed during submission that patient consent has been signed and collected in accordance with the journal's patient consent policy.

## Data Availability

Data available on request from the authors.
